# A strategy to assess spillover risk of bat SARS-related coronaviruses in Southeast Asia

**DOI:** 10.1038/s41467-022-31860-w

**Published:** 2022-08-09

**Authors:** Cecilia A. Sánchez, Hongying Li, Kendra L. Phelps, Carlos Zambrana-Torrelio, Lin-Fa Wang, Peng Zhou, Zheng-Li Shi, Kevin J. Olival, Peter Daszak

**Affiliations:** 1 EcoHealth Alliance, New York, NY USA; 2 Department of Environmental Science and Policy, George Mason University, Fairfax, VA USA; 3 Programme in Emerging Infectious Diseases, Duke-NUS Medical School, Singapore, Singapore; 4 Wuhan Institute of Virology, Chinese Academy of Sciences, Wuhan, China

**Keywords:** Ecological modelling, Ecological epidemiology, Viral infection

## Abstract

Emerging diseases caused by coronaviruses of likely bat origin (e.g., SARS, MERS, SADS, COVID-19) have disrupted global health and economies for two decades. Evidence suggests that some bat SARS-related coronaviruses (SARSr-CoVs) could infect people directly, and that their spillover is more frequent than previously recognized. Each zoonotic spillover of a novel virus represents an opportunity for evolutionary adaptation and further spread; therefore, quantifying the extent of this spillover may help target prevention programs. We derive current range distributions for known bat SARSr-CoV hosts and quantify their overlap with human populations. We then use probabilistic risk assessment and data on human-bat contact, human viral seroprevalence, and antibody duration to estimate that a median of 66,280 people (95% CI: 65,351–67,131) are infected with SARSr-CoVs annually in Southeast Asia. These data on the geography and scale of spillover can be used to target surveillance and prevention programs for potential future bat-CoV emergence.

## Abstract (web summary)

Coronaviruses may spill over from bats to humans. This study uses epidemiological data, species distribution models, and probabilistic risk assessment to map overlap among people and SARSr-CoV bat hosts and estimate how many people are infected with bat-origin SARSr-CoVs in Southeast Asia annually.

## Introduction

Emerging coronaviruses (CoVs) of wildlife origin have significantly disrupted global health security and economies during the last two decades^1,2^. Severe acute respiratory syndrome (SARS) and Middle East respiratory syndrome (MERS) CoVs caused significant human morbidity and mortality in 2002 and 2012 respectively^3,4^. Swine Acute Diarrheal Syndrome CoV caused substantial mortality in pigs in southern China during 2016 and 2019^5,6^. The emergence of SARS-CoV-2 in 2019 led to the current COVID-19 pandemic that has caused millions of cases and deaths, with economic loss likely to be in the tens of trillions of US dollars^2,7^. Efforts to increase preparedness and improve surveillance for emerging coronaviruses therefore represent a priority for global health programs^8^.

Phylogenetic analysis suggests that SARS-CoV, MERS-CoV, SADS-CoV, and SARS-CoV-2 originate within CoV lineages from bat reservoir hosts^5,9–11^. The initial spillovers of SARS and MERS into human populations are thought to have occurred via intermediate hosts (palm civets and dromedary camels, respectively^12,13^). However, the role of civets in the emergence of SARS is uncertain, and other bat SARSr-CoVs can directly infect human cells, including airway epithelial cells, and thus have potential to spill over directly from bats to humans^14–16^. In support of this idea, serological evidence of prior infection with SARSr-CoVs was found in communities living near bat populations in China prior to the emergence of COVID-19, including in people who reported no contact with SARSr-CoV intermediate hosts^17,18^. Direct bat-to-human spillover events may occur more frequently than has been reported, but go unrecognized because they cause mild symptoms, cause symptoms similar to those of other infections, result in small case numbers, or lack sustained chains of human-to-human transmission. However, every wildlife-to-human spillover event represents an opportunity for viral adaptation that could permit human-to-human spread^19–22^. Estimating the extent of these undetected spillovers could therefore be important for identifying the likelihood of future epidemics or pandemics.

Surveys of bats in China have revealed high diversity of SARSr-CoVs, and often high infection prevalence (5–10%) in rhinolophid and hipposiderid species that are widely distributed and abundant, with varying resilience to habitat perturbation and many synanthropic species (having contact and often interactions with human populations)^23–25^. Many of the bat species and genera known to harbor these β-CoVs occur in Southeast Asia, a hotspot of bat diversity with 441 species reported, 115 (around a quarter) of which are rhinolophids or hipposiderids^26^. Diversity of SARSr-CoVs is also likely high in this region^23^, but may be underestimated as CoV research effort in China appears to have been far more intense than in nearby Southeast Asian or South Asian countries^27^ (see Methods and Supplementary Fig. 1). Furthermore, many of these less well-sampled countries are undergoing dynamic social and environmental changes correlated with zoonotic emergence (e.g., rapid human population growth, movement of rural residents to urban centers, extensive wildlife farming and trade, rapid land conversion from forested habitats to agricultural land), and thus might represent hitherto unreported hotspots for coronavirus spillover^23,28–33^.

In this study, we use host distribution modeling as well as human behavioral and epidemiological data to estimate the geographic distribution of SARSr-CoV bat hosts, and the likely rate of zoonotic spillover in China, South and Southeast Asia. Our results provide detailed estimates of the distribution of bat hosts of SARSr-CoVs and suggest that the magnitude of SARSr-CoV spillover from bats to humans may be substantially underestimated. Our approach provides proof of concept for assessing the rate of zoonotic spillover and identifies key geographic areas that can be prioritized for targeted surveillance of animals and humans. Given the challenges of identifying the origins of COVID-19 and pathways by which SARS-CoV-2 spilled over to people^34,35^, our results may also aid efforts to identify the geographic sites where spillover first occurred.

## Results

We assembled a list of 26 known SARSr-CoV bat host species that occur in our geographic region of interest (a broad region including parts of South Asia, China and Southeast Asian countries; see Methods for a list of countries and administrative regions included in the analysis). Host species were mainly members of *Rhinolophidae* and *Hipposideridae* families, but also included two members of *Molossidae* and one member of *Vespertilionidae* (Supplementary Table 1). We derived the area of habitat (AOH)^36^ for each species by refining the IUCN geographic range of each species according to habitat suitability, elevation limits, and the boundaries of our region of interest. Removing unsuitable areas within the IUCN geographic ranges greatly reduced the size of species distributions. For example, the reduction in area from the original IUCN range to the more refined AOH ranged from 42% for *Rhinolophus malayanus* to almost 100% for *R. hipposideros*, with a median of 65% reduction across all species (Supplementary Fig. 2). The reduction in area was 55% for *R. rex*, the only species in our list assessed as endangered by the IUCN Red List.

We validated species AOHs using cleaned occurrence records downloaded from the Global Biodiversity Information Facility (GBIF; see Methods for details of data cleaning and validation). After data cleaning, no occurrence records remained for *R. hipposideros*, while 1–621 (median = 42) occurrence points remained for all other species (Supplementary Table 2). In validating each species’ AOH with GBIF occurrence points, we found among species with ≥1 occurrence point, the median percent of points (buffered by 5 km) that overlapped a species’ AOH was 62% (Supplementary Table 2). Among species with at least 40 occurrence points (*n* = 15), the median overlap was 76%. Overlap was ≥80% for five species: *R. luctus* (63/69, 91%), *R. creaghi* (37/41, 90%), *R. malayanus* (45/52, 87%), *R. shameli* (35/42, 83%), and *Hipposideros galeritus* (64/80, 80%).

**Fig. 1:**
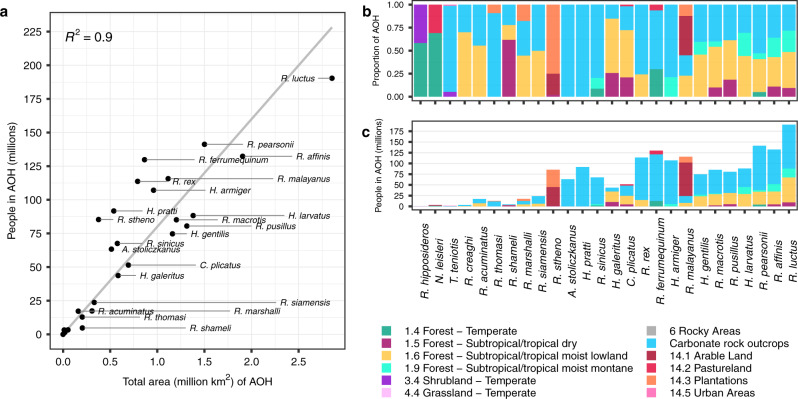
Relationships among area of habitat (AOH) size, number of people, and habitat proportions. **a** Scatterplot showing the total number of people living in each AOH versus the total area, for each SARSr-CoV bat host species. A best-fit line was fit through the origin, for which the R^2^ is displayed. The four unlabeled species at the bottom left corner are (left to right): *R. hipposideros*, *N. leisleri*, *T. teniotis*, and *R. creaghi*. **b** Proportion of each habitat type (by area) within species AOHs. **c** People living in the AOH of each species, separated by habitat type. For **b** and **c**, species are listed in order of increasing AOH size, and the species order is the same in both panels. Genus abbreviations in all panels: *A*. = *Aselliscus*, *C*. = *Chaerephon*, *H*. = *Hipposideros*, *N*. = *Nyctalus*, *R*. = *Rhinolophus*, *T*. = *Tadarida*.

The size of individual species AOHs varied widely, but generally, the largest AOHs encompassed the most people (Fig. 1a). For example, the AOH of *R. luctus* was the largest of all species, covering ~2.9 million km^2^, and encompassed ~190 million people, the most of any species. Only three other species had AOHs encompassing more than 125 million people (*R. pearsonii*, *R. affinis*, and *R. ferrumequinum*), with the AOHs of *R. affinis* and *R. ferrumequinum* both encompassing ~130 million people despite the AOH of *R. ferrumequinum* being less than half the size of that of *R. affinis*. Fewer than 5 million people live in the AOHs of *R. hipposideros*, *Nyctalus leisleri*, *Tadarida teniotis*, *R. creaghi*, and *R. shameli*. Two species had AOHs with more limited area but relatively high human population density: *N. leisleri* (~262 people/km^2^) and *R. stheno* (~226 people/km^2^; Fig. 1a, Supplementary Fig. 3). Within species AOHs, forest habitats and carbonate (limestone) rock outcrops—used as a proxy for cave distribution—typically comprised the largest proportion of suitable habitat (Fig. 1b). Four species were highly reliant on carbonate rock outcrops (i.e., their AOHs were comprised of >90% of this habitat type): *T. teniotis*, *R. thomasi*, *Aselliscus stoliczkanus*, and *H. pratti*. Only *R. stheno* and *R. malayanus* had AOHs comprising >50% artificial habitats, including plantations and arable land. Examining the number of people in the habitat type of each species revealed that people tended to be over-represented in carbonate rock outcrop areas compared to forest areas, relative to the size of these habitats (Fig. 1c). For example, carbonate rock outcrops comprised 28%, by size, of the AOH of *R. luctus*, but represented 54% in terms of people living in this habitat type; similarly, these percentages were 53% and 73% for *R. pearsonii*. Arable land also appeared overrepresented in terms of the number of people living in this habitat versus its proportion by size, as observed for *R. stheno* and *R. malayanus*.

**Fig. 2:**
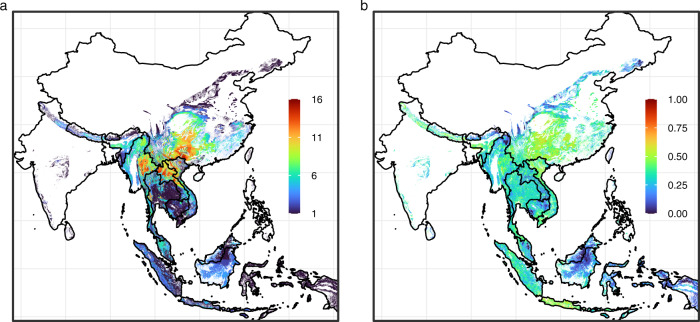
Hotspots of SARSr-CoV bat host species richness and human overlap in Southeast Asia. **a** Species richness of SARSr-CoV bat host species in Southeast Asia, created by overlaying area of habitat maps for all 26 SARSr-CoV bat host species known for this region. **b** Relative bat-human overlap: bat host species richness multiplied by human population count. Values were ln(*x* + 1) transformed and then normalized to a 0–1 scale. For both panels, redder colors indicate larger values and bluer colors indicate smaller values.

The consensus area of all SARSr-CoV bat host species, created by overlaying the 26 species AOHs, comprised ~5.1 million km^2^. We calculated that ~499 million people live within this consensus area, which covered most of Lao PDR, Cambodia, Thailand, Vietnam, Nepal, Bhutan, peninsular Malaysia, Myanmar, southeast China, and the western islands of Indonesia (Fig. 2a). Bat species distribution was patchier in India, Sri Lanka, East Malaysia, and the Philippines. Species richness ranged from 1–16 species, with the highest richness of SARSr-CoV bat host species in southern China, eastern Myanmar, and northern Lao PDR (Fig. 2a). When we visualized areas with both high host richness and large human populations (bat-human overlap), southern China remained a hotspot, while other areas emerged as important because of their high human population sizes (e.g., Java, parts of northern India, parts of Myanmar; Fig. 2b).

**Fig. 3:**
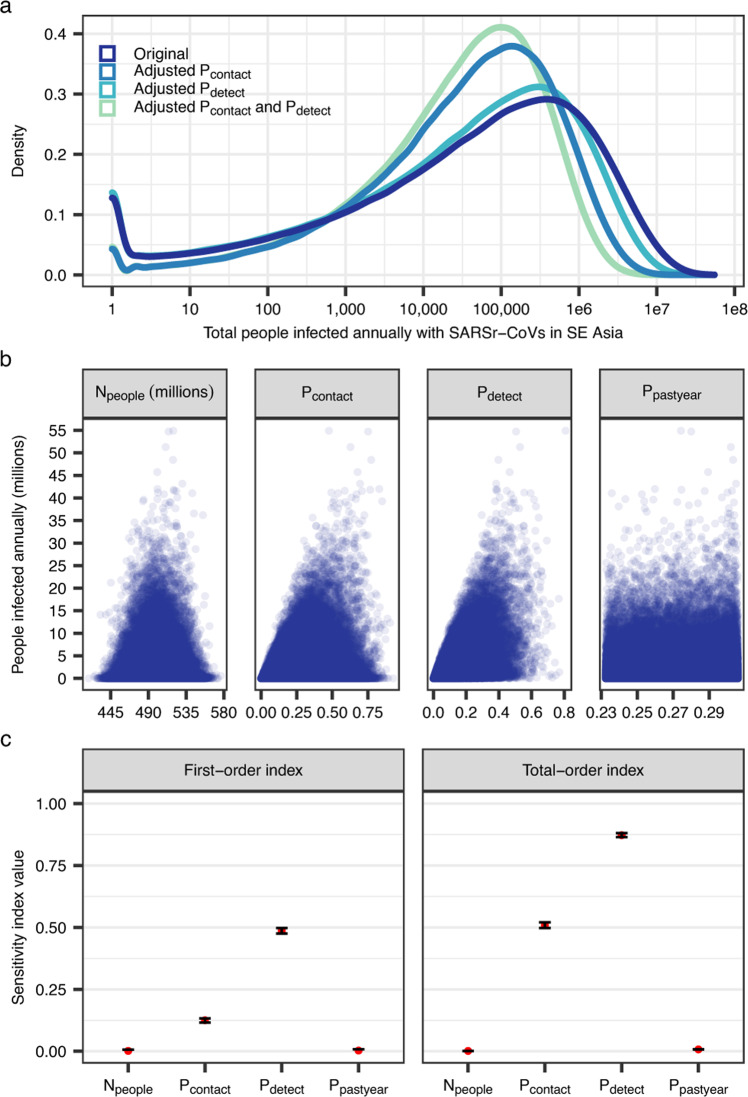
Spillover simulations and sensitivity analyses. **a** Density plots of the estimated total number of people in Southeast Asia infected with SARSr-CoVs by bats each year. Line colors represent scenarios exploring how estimated spillover changes if one or both of two parameter distributions (P_contact_ and P_detect_) are refit after excluding the highest estimates of these parameters gathered via literature searches (see Methods for details). Note that the x-axis is on a log_10_ scale. **b** Estimated total number of people in Southeast Asia infected with SARSr-CoVs by bats each year, plotted as a function of four input variables. Values correspond to the original scenario (i.e., no adjusted parameters). **c** Sobol sensitivity indices, indicating the amount of variance in the outcome due to each input on its own (first-order index) and the amount of variance in the outcome due to each input including interactions with other inputs (total-order index). Sobol indices were calculated with two random samples of 200,000 points each. Red dots represent calculated index values and error bars represent 95% confidence intervals. Values correspond to the original scenario (i.e., no adjusted parameters).

After calculating the number of people living in the consensus area of SARSr-CoV bat host species, we incorporated data from the literature on human-bat contacts, viral seroprevalence among humans reporting bat contact, and human SARS antibody duration to estimate spillover risk of SARSr-CoVs in Southeast Asia (see Methods for details). For this analysis, we define spillover risk as the annual number of people infected by SARSr-CoVs via bat-to human transmission events. This definition draws from the work of Hosseini and colleagues^37^, who define risk as equal to hazard x exposure x vulnerability, where a hazard is a potential source of harm from a microbe, exposure is the likelihood of contact between humans and hazards, and vulnerability is the chance of a hazard causing harm, given exposure. Here, the hazard is a bat belonging to a species known to host SARSr-CoVs, exposure is the likelihood of contact between a person and a bat, and vulnerability is the chance that a human-bat contact event results in a human infection that produces detectable antibodies. Note that we do not consider whether a human infection will lead to sickness or an outbreak, because these outcomes depend on a range of other factors including viral genotype and phenotype, host susceptibility (e.g., age, predisposing conditions), and population-level factors that would affect the ability of a pathogen to spread; these are outside the scope of the current study. We estimated that within the consensus area of SARSr-CoV bat host species, a median of 66,280 people (95% confidence interval for the median: 65,351–67,131; overall range: 1–54,905,544; interquartile range: 3,250–519,557) are infected with SARSr-CoVs annually in Southeast Asia (Fig. 3a).

Sensitivity analyses (see Methods) indicated that two parameters—the probability of human contact with a bat (*P*_*contact*_), and the probability of antibody detection given contact with a bat (*P*_*detect*_)—primarily contributed to variance in the outcome (Fig. 3b, c). Specifically, *P*_*detect*_ contributed most to variance in the outcome (first-order sensitivity index: 0.486; 95% CI: 0.475–0.496; total-order sensitivity index: 0.873; 95% CI: 0.864–0.881), while *P*_*contact*_ contributed moderately to variance in the outcome (first-order sensitivity index: 0.125; 95% CI: 0.117–0.134; total-order sensitivity index: 0.509; 95% CI: 0.499–0.519). We therefore explored how refitting the distributions for these parameters (Supplementary Fig. 4), after excluding the highest estimates of *P*_*contact*_ and *P*_*detect*_ found in our literature searches, changed our estimates of spillover (Fig. 3a). Refitting only the *P*_*contact*_ distribution lowered the median number of people infected to 53,290 (95% CI: 52,621–53,957; range: 1–33,119,404; interquartile range: 2,817–375,452), while refitting only the *P*_*detect*_ distribution lowered the median to 49,599 people (95% CI: 49,151–50,095; range: 1–20,694,204; interquartile range: 6,262–234,140). When both distributions were refit, the median number of people infected was 38,910 (95% CI: 38,551–39,268; range: 1–11,653,741; interquartile range: 5,538–163,053).

## Discussion

Our paper reports an analytical framework to assess SARSr-CoV spillover risk in a region that includes the site of the first detected spillover of SARS-CoV, and likely of SARS-CoV-2. We first provide detailed maps of SARSr-CoV bat host richness and bat-human overlap in South and Southeast Asia. Using information gathered from the literature on human-bat contacts, viral seroprevalence among humans with bat contact, and human SARS antibody duration, we then provide the first estimates, to our knowledge, of the number of people infected by SARSr-CoVs annually. The analytical framework, maps, and other results produced here may assist public health measures by identifying regions for targeted surveillance and early detection of ongoing spillover events, for viral discovery programs to identify novel bat-CoVs, and for COVID-19 origin tracing. All of these are key goals for pandemic preparedness and prevention^38–41^, and if used to target future surveillance and disease control, may help to reduce the possibility of future COVID-like outbreaks.

Our analysis identifies regions in southern China, northeastern Myanmar, Lao PDR, and northern Vietnam as having the highest diversity of SARSr-CoV bat host species, in concordance with other recent efforts to map the distribution of SARSr-CoV bat host species^42^. These hotspots of SARSr-CoV bat reservoir host diversity may be particularly fruitful sites for viral discovery of novel SARSr-CoVs, assuming that viral diversity scales with host species diversity^43^. This finding supports conclusions from prior phylogenetic analyses that particularly diverse SARSr-CoV lineages are found in southern China^23^. Our results also help explain the recent identification of multiple strains of SARSr-CoVs in southern China^33,44,45^ and southeast Asian countries^30^, despite small sample sizes. Our findings suggest that less intense sampling in countries bordering southern China (Supplementary Fig. 1) could have led to an underestimate of the diversity of these viruses there^27,32^. Given that the bat species known to host the closest relatives of SARS-CoV-2 are found in this region, our species richness map may also guide efforts to identify the viral clade from which a progenitor virus emerged^23,33,35^.

The map of bat-human overlap reveals hotspots in southern China and bordering countries, but also in the populous regions of Indonesia. This map may be useful in targeting surveillance to identify SARSr-CoV spillover events in people, including syndromic surveillance for SARS- or COVID-like respiratory disease in communities within these hotspots. This has been proposed previously as a tool for proactive surveillance for novel infections that could become standardized across emerging disease hotspots^38,43^. In this case, surveillance in clinics for severe/acute respiratory illness, influenza-like illness, and fevers of unknown origin would capture pneumonia or rapid-onset respiratory distress typical of SARS and COVID-19 patients, and likely to be expected from other novel bat-SARSr-CoV spillover infections^46^. More common infections such as influenza and bacterial or fungal pneumonia could be ruled out relatively easily^47^. The bat-human overlap map may also provide guidance for studies of where initial spillover of the progenitor of SARS-CoV-2 may have occurred, although epidemiological and other data suggest this would most likely have been in China or neighboring countries^34,35^.

Our estimate that a median of ~66,000 people are infected with SARSr-CoVs each year in Southeast Asia suggests that bat-to-human SARSr-CoV spillover is common in the region, and is undetected by surveillance programs and clinical studies in the majority of cases. While our results suggest significant levels of spillover, many of the diverse viral strains that infect people in the region each year may not be able to replicate well in people, cause illness, or be transmitted sufficiently among people to cause an outbreak. This has been shown in theoretical models of disease emergence^21,48^, and supports earlier evidence from studies of non-human primate virus spillover^49^, that cross-species transmission of novel animal-origin viruses is not the rate-limiting step in pandemic viral emergence. However, given the relatively large number of people likely to be infected each year with bat-CoVs, it is plausible that illnesses or clusters of cases due to novel bat-CoV infection occur regularly within the region, and are either not reported or are missed by clinical surveillance. Evidence of underreporting has been demonstrated for other bat-origin viral infections. For example, targeted syndromic surveillance of encephalitis patients in a small number of clinics in Bangladesh showed that Nipah virus causes outbreaks annually with an overall mortality rate of ~70%, despite it only recently being reported from the country^50^. Efforts to increase surveillance for novel SARSr-CoVs (and other emerging viruses) in clinical cohorts, particularly using syndromic surveillance, may identify the rate of missed cases and pre-empt larger scale outbreaks. Estimating the true rate of spillover of previously unknown, potentially zoonotic animal-origin viruses is difficult without serological or genomic surveillance data. For most viruses, the duration of infection in humans is relatively short (e.g., the infectious period for COVID-19 is 2–3 weeks^51^), and if spillover is rare, PCR surveillance is unlikely to give valuable data on spillover rates due to lack of positives. One exception is for viruses with long infectious periods such as lentiviruses and some retroviruses; a previous genomic surveillance study of wildlife hunters in Africa was able to find 10/1099 (0.9%) prevalence of non-human primate origin simian foamy viruses^52^. Because detectable antibodies or T cell responses are more long-lived, serological surveys or activated T cell testing represent more valid strategies to estimate spillover rates.

Our calculation of undetected spillover represents the first published attempt, to our knowledge, to identify the spillover risk of SARSr-CoVs from bats to people. It relies on a number of input variables, and we attempted to account for uncertainty (e.g., potential inaccuracy of serological tests) and variation (e.g., differences in human behavior due to gender, occupation, cultural norms) associated with these inputs by assigning a probability distribution to each variable, based on data gathered from the literature. We discuss these data, and some of the assumptions we make in using them, in more detail below.

Human-bat interactions likely vary widely across our study region, and are influenced by a variety of social, ecological, and economic factors at individual and community scales and beyond^53–55^. We therefore performed a systematic literature search to gather estimates of human-bat contact prevalence reported in previous surveys and ethnographic investigations. Our literature search revealed that limited data exist on human contact with bats in Southeast Asia, and that most studies of human-bat contact have targeted rural communities where bats are present and contact events could occur due to occupational or environmental exposure. As a result, estimates of human-bat contact reported in these studies might be higher than would be found in other human populations (i.e., more urban communities). However, we consider it plausible that urban residents face comparable levels of contact with bats as rural residents, though the interfaces may be different. For example, a recent study showed that rural and urban human populations in China displayed similar patterns of contact with wild animals, including bats^56^. Although some dense urban areas may provide less suitable roosting habitat for many bat species as compared to rural or peri-urban areas (meaning urban residents could be less likely to have bats in their home, visit caves, or hunt bats), several synanthropic bat species are able to thrive in human-developed landscapes^57^. Of the host species considered in this study, IUCN habitat type 14.5 (Urban areas) is considered suitable for two species, while other artificial terrestrial habitat types (14.1: Arable land, 14.2: Pastureland, 14.3: Plantations, 14.4: Rural gardens, 14.6: Subtropical/tropical highly degraded former forest) are suitable for 2–5 species each (Supplementary Table 1). Further, interfaces such as the wildlife trade—including physical markets and the supply chain—represent a major potential exposure pathway for people living in more densely populated urban settings. For instance, a study of the bat trade in Indonesia demonstrated that bats are harvested in rural areas but are directly transported to more urban and developed areas with more buying power^58^. Other work has found that wildlife (*Rattus* and *Bandicota* spp.) CoV prevalence increases along the distribution chain (i.e., from traders to large markets to restaurants), and suggested that end consumers face “maximal risk”^59^. Given this evidence, and in the absence of better data on contact with bats for urban residents, we believe it is reasonable to apply the bat-human contact rates generated from our literature search across our study region.

In determining the distribution for *P*_*detect*_ (the parameter describing the probability that a human-bat contact leads to a serologically detectable human infection), we incorporated literature-gathered seroprevalence estimates from people exposed to CoVs and other directly transmitted RNA viruses. We believe we are justified in using non-CoV data in our analyses for several reasons. First, there are very few pre-COVID human serosurveys that specifically tested for SARSr-CoV exposure^17,60^, and we wanted to parameterize our spillover estimate with a wide range of possible values for infection and seroconversion using other RNA viruses that are known or likely able to be transmitted via direct exposure to bats or bat excreta. We acknowledge that there may be substantial variation in the ability of different SARSr-CoVs or other bat-harbored viruses to cause infection and seroconversion in humans; however, we know little about the large diversity of viral strains circulating in bat populations and the host cellular and immunological factors that could influence seroconversion after exposure for most viruses^61^. Indeed, many seroprevalence estimates reported in the literature may be an underestimate. For example, some individuals subclinically infected by SARS-CoV-2 may clear infection prior to production of antibodies^62^ and some bat SARSr-CoVs may require limited evolutionary adaptation before antibody responses can be produced (e.g., data from RaTG13 binding^63^). A study of memory T-cells in people in Singapore detected SARS-CoV-2-specific interferon-γ responses in approximately half (19/37) of people negative for SARS-CoV-1 or SARS-CoV-2 infection, and suggested unknown, potentially animal-origin CoVs could induce cross-reactive SARS-CoV-2 T cells^64^. Data from serological analysis also demonstrates high seroprevalence in Lao PDR. Specifically, Virachith and colleagues detected SARS-CoV-2-specific anti-N and anti-S antibodies in 5.3% and 1.1%, respectively, of human serum samples collected in 2018 (i.e., pre-COVID-19) in Lao PDR^60^. The authors also tested serum samples collected during the pandemic (August–September 2020) and detected antibodies in 5.2% (anti-N) and 2.1% (anti-S) of the general public, and in 20.3% (anti-N) and 6.8% (anti-S) of bat/wildlife contacts (bat guano collectors, wildlife animal vendors, and family members of bat contacts, catchers or guano collectors). The authors noted that high seroprevalence may also reflect the frequent trade in bush meat and low biosafety awareness reported in earlier surveys in Lao PDR^60,65^.

**Fig. 4:**
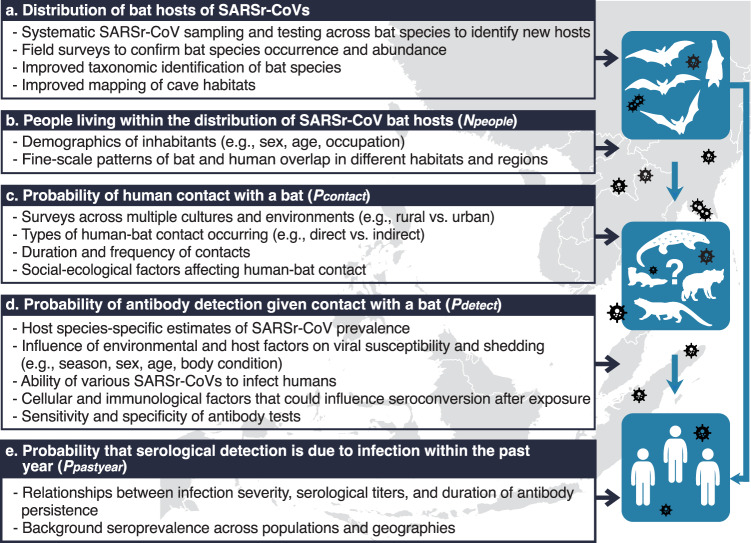
Key data inputs and future research needs to improve estimates of SARSr-CoVs spillover from bats to humans. Additional data inputs are organized according to steps in our probabilistic risk assessment to improve our understanding of: **a** wildlife reservoir host (bat) distribution, **b** people overlapping with bat hosts, **c** probability of human-bat contact, **d** probability of detecting antibodies given contact, and **e** dynamics of antibody response in individuals.

Previous authors have commented on the need to develop approaches to assessing zoonotic spillover risk that explicitly incorporate uncertainty^66^, and we recognize that further data would likely help refine estimates of SARSr-CoV spillover rates (Fig. 4). Perhaps most critical is a lack of information on the role of intermediate hosts in the emergence of SARSr-CoVs. It has been postulated that civets and other commonly farmed and traded mammalian species played a role in the emergence of SARS-CoV by acting as efficient amplifier hosts within wildlife farms and markets^12,67–70^. SARS-CoV-2 can infect civets, raccoon dogs, and other mammals commonly farmed and traded for food in the region^71,72^. SARS-CoV-2 has also caused significant outbreaks in animals bred for fur (e.g., mink, raccoon dogs) that have in some cases led to transmission to people^73,74^. These and other data led an international team to conclude that the most likely pathway for COVID emergence was from bats to people through a farmed mammalian intermediate host^34^. Accurate data are not available on the number of wildlife farms and potential intermediate hosts bred each year, or on the market systems that supply live animals into cities within China and Southeast Asia. It was estimated that 14 million people were employed in the wildlife farming system within China alone during 2016, in an industry worth $77 billion annually^75,76^. Additionally, some of these potential intermediate hosts occur naturally in the region (e.g., pangolins, civets), and other livestock species that are common (e.g., pigs, cattle, rabbits) are susceptible to SARSr-CoVs either naturally or experimentally^72^. Spillover of SARSr-CoVs may therefore be substantially skewed to people who have high contact with these species, and this would likely have been missed in the serological surveys upon which our analyses are currently based. Thus, better estimates of the role of farmed and traded intermediate hosts are likely to substantially increase the estimated spillover rates of SARSr-CoVs across the region.

Other data are needed to improve and help validate our estimates from limited knowledge. Useful research could include bat surveys to identify contemporary, accurate species presence records, and sampling a range of bat species for SARSr-CoVs to identify previously unknown SARSr-CoV hosts (Fig. 4a). Statistical models can inform prioritization of species to sample to optimize viral discovery^77^. Further data are also needed on the role of host biology and ecology in spillover: for instance, improved species-specific estimates of SARSr-CoV prevalence, and the effects of environmental factors (e.g., location, season, year) and host factors (e.g., sex, age, body condition, reproductive status) on viral shedding^78–81^ (Fig. 4d). As described previously, there is a need to better understand how and why people and bats come into contact in regions of overlapping occurrence (Fig. 4b, c). Crucial immunological information includes expanded survey data on the background seroprevalence of bat SARSr-CoVs in people across different populations and geographies, improved understanding of factors that could influence seroconversion after exposure, and studies of how serological titers and duration of antibody persistence relate to severity of infection (Fig. 4d, e). Finally, it is also possible that taxonomic errors have occurred in the data we analyzed. Taxonomic standards in viral discovery and surveillance studies vary widely, and point to a need for better taxonomic training of field teams, standardized DNA barcoding of hosts, collection of voucher specimens, and closer collaboration among disease ecologists, virologists, field biologists and taxonomists^66,82,83^.

Our refinement of species ranges has produced a detailed picture of SARSr-CoV bat host species potential occurrence. The AOHs we produced may be useful for targeting surveillance to key species. For example, only a few species’ AOHs contained a sizable proportion (≥25%) of one or more artificial habitats (Fig. 1b): *R. malayanus* (arable land, plantations), *R. stheno* (plantations, arable land), and *N. leisleri* (pastureland). These species may be more important as spillover hosts considering that these modified landscapes have a higher opportunity for human-wildlife contact and are known to have heightened diversity of other zoonotic disease hosts^84^. Cave habitats were classified by the IUCN as suitable for nearly all species in our analyses, and carbonate rock outcrops (used here as a proxy for caves) made up a large proportion of species AOHs. Public presence in caves and livelihoods or occupational activities involved in bat guano collection are likely particularly hazardous given these findings^85–88^. Our validation process indicated good agreement between species AOHs and GBIF occurrence data, suggesting that the maps we generated accurately reflect species presence for occurrence records collected after 1990. We note that external forces could drive bats away from otherwise suitable habitats; for example, many cave-dwelling bats face disturbance in the forms of tourism, guano harvesting, vandalism, land use change, and more^89–91^. Limitations of AOH maps include the potential inaccuracy of the IUCN species ranges, habitat suitability assignments, and elevation limits^36,92^. All but one of the species in our dataset were last assessed by the IUCN in 2016 or more recently (*A. stoliczkanus* was last assessed in 2008); in future, revised AOHs can be developed based on new IUCN range data. The map of terrestrial habitat types used in our analyses^93,94^ is not a perfect representation of habitat, but is the most accurate and detailed analysis of its kind to date. In particular, the map did not include caves, an important habitat type for many bat species. We used carbonate rock outcrop data as a proxy for cave distribution and this should be ground-truthed. Finally, though grid cells are assigned to one dominant habitat class, at a fine-scale level they are comprised of various microhabitats which may be more or less attractive to wildlife.

Our analytical framework provides a strategy that has potential for improving preparedness for emerging diseases and pandemics. However, it is important to recognize that our results specifically identify spillover risk, which we defined as the annual number of people infected by SARSr-CoVs via bat-to-human transmission events. Our analyses do not assess the impact of spillover, the severity of disease that results (if any), the ability of infections to lead to human-to-human (community) transmission, or the likelihood of a regional outbreak or a pandemic^37^. Furthermore, we did not account for differences in vulnerability among populations, communities, or individuals. However, our analyses have public health value in that the maps can be used to conduct more cost-effective field surveys for viral discovery programs, guide human surveillance to identify clusters of cases of a new CoV infection earlier and help prevent spread, and guide targeted epidemiological studies of small-scale processes that increase spillover risk^95–97^. Our analysis pipeline and framework are based on open-source code and can therefore serve as a resource to update and modify spillover risk maps and estimates as new data become available. Finally, our framework can be rapidly adapted for spillover risk assessment of other viral groups, such as the HKU-2/SADS-CoV α-CoVs that have recently been found able to infect primary human airway epithelial cells in vitro, and therefore pose a heightened spillover risk, or any of the other ~25 viral families that include known zoonoses^43^.

## Methods

All analyses were performed in the R statistical environment v. 4.0.3 (R Core Team 2020).

### Estimation of coronavirus research effort

To assess CoV research effort in Southeast Asia, we used the R package rentrez v1.2.3^98^ to query the PubMed database for the number of publications from each country in Southeast Asia (see below), as of November 17, 2021, using the search string: (“bat” OR “bats” OR “Chiroptera”) AND (“coronavirus” OR “coronaviruses”)^83^. We visualized a map of CoV research effort using the R package rworldmap v1.3–6^99^.

### Compilation of SARSr-CoV bat host data

We identified all bat species from which molecular evidence of SARSr-CoV infection had been reported, and for which associated sequence confirmation data were available. We supplemented a recently compiled list^100^ with hosts listed in other more recent publications^23,30,32,44,101,102^. We refrained from including bat species that have been predicted, but not confirmed, to be SARSr-CoV hosts. We considered *R. paradoxolophus* to be a subspecies of *R. rex*^103^. We excluded *R. monoceros* because, although recent work has retained it as a distinct species^104^, no recent assessment of the species has been published by the IUCN Red List of Threatened Species. *H. pomona* and *H. gentilis* were split in 2018, with *H. pomona* restricted to a small area in southern India while *H. gentilis* is broadly distributed across Southeast Asia^105^. Although Latinne and colleagues^23^ listed *H. pomona* as a host species, because the field sampling was conducted in China, we substituted *H. gentilis* in our analyses.

We included only bat hosts with geographic distributions either partially or entirely within Southeast Asia (see Supplementary Table 1 for the finalized list of SARSr-CoV bat hosts). The area covered by the following countries and administrative regions is included in our analysis extent: Bangladesh, Bhutan, Brunei, Cambodia, China, Hong Kong SAR, Macao SAR, India, Indonesia, Lao People’s Democratic Republic, Malaysia, Myanmar, Nepal, the Philippines, Singapore, Sri Lanka, Thailand, Timor-Leste, and Vietnam. Note that while we use the term Southeast Asia, the region we considered has broader range than is used for political definitions of the region and includes China and parts of South Asia, due to the extensive range of some of the bat host species. All analyses described below were restricted by the regional boundaries of Southeast Asia as defined above.

### Calculation and validation of host area of habitat

To assess SARSr-CoV bat host distribution and richness across our study area, we derived the area of habitat (AOH) for each species. AOH describes the habitat suitable for a species within its range according to the species’ habitat preferences and elevation limits^36^. Previous validation of AOH maps with species occurrence data has demonstrated their high accuracy; for example, ~92% (241/263) of terrestrial mammal species AOHs predicted species point localities better than their IUCN geographical range^106^. Determining a species AOH does not rely on presence data, making it useful when assessing species with few occurrence records (as was the case for many species in our analysis; see Results).

To derive the AOH for each species, we downloaded its geographic range from the IUCN Red List of Threatened Species^26^ (last update: March 25, 2021), overlaid it onto a raster map of terrestrial habitat types^93,94^, and selected areas of the map that occurred within its range. We then selected areas of suitable habitat for each species. Specifically, using the IUCN Red List data, we assessed if each of 22 habitat types was suitable for each bat host species. According to the IUCN Habitats Classification Scheme, a designation that a habitat is suitable means “the species occurs in the habitat regularly or frequently”^107^. The 22 habitat types we examined included various forest habitats, shrublands, rocky areas and caves, and artificial habitats, chosen from a larger list of ~100 habitat types^107^ as being influential in determining the occurrence of one or more target host species (Supplementary Table 1). A global land cover map of these habitat types^93,94^ included 18 of the 22 target habitats. Because two habitat types (7.1. Caves and Subterranean Habitats (non-aquatic)—Caves; 7.2. Caves and Subterranean Habitats (non-aquatic)—Other subterranean habitats) were not included in the global land cover map, but were suitable for many host species, we supplemented the land cover dataset with the World Map of Carbonate Rock Outcrops v3.0^108^ as a proxy for karst landscape and cave distribution. This dataset comprises two layers, one with areas of continuous carbonate rocks and another with abundant but not continuous rocks; we used the former for our analyses. We rasterized the carbonate rock outcrop shapefile using the R package fasterize v1.0.3^109^ and combined this with the land cover raster. When restricting species IUCN ranges by suitable habitat, we used the combined land cover/carbonate rock raster if a species was found in habitat type 7.1, and the land cover-only raster if a species was not found in this habitat type. The other two habitat types not represented on the land cover raster were only suitable for 1–2 host species (i.e., 14.6. Subtropical/Tropical Heavily Degraded Former Forest; 15.8. Seasonally Flooded Agricultural Land); therefore, we did not attempt to find other data to use as a proxy for these habitats.

Finally, we overlaid elevation data (Shuttle Radar Topography Mission data obtained with the getData function of the R package raster v3.4–5^110^) onto each species’ range and habitat map and selected areas that fell within a species’ elevation limits (Supplementary Table 1); these remaining areas represented the species’ AOH. Using the area function of the raster package^110^, we calculated the size (in km^2^) of each species’ AOH and compared it to the size of its original IUCN range.

We validated the AOH of each species using occurrence data downloaded from GBIF using the R package rgbif v3.6.0^111–113^. We cleaned GBIF data by removing records with inaccurate or imprecise coordinate data (i.e., no coordinates, identical longitude and latitude, coordinates of 0, coordinate uncertainty >35 km, coordinates in the ocean, country–coordinate mismatch), records from areas outside our geographically defined region, records of absence rather than presence, and records with a Basis of Record other than human observation, machine observation, observation, material sample or preserved specimen. We removed records within 5 km of country capitals and within 1 km of country/province centroids (as coordinates are likely to be assigned to capitals and centroids in the absence of detailed location information), and within 100 m of biodiversity institutions (as these coordinates would represent the physical location of a specimen, rather than its capture location). We removed records before 1991, as older records tend to have less precise location data^114^ and could reflect species ranges that have since shifted. Within each species, we removed records with duplicate coordinates. Cleaning was facilitated with the R package CoordinateCleaner v2.0–18^115^. For each species, we buffered each of its occurrence points by a five-kilometer radius and calculated the percent of buffered points that overlapped the species’ AOH.

### Estimation of bat-human overlap, bat species richness, and habitat proportions

To identify regions where human populations might be exposed directly or indirectly to SARSr-CoV bat hosts, we overlaid human population count data on each host species’ AOH. We used a 1-km resolution raster of 2020 population count data from WorldPop^116^ and resampled it with bilinear interpolation using the R package gdalUtils v2.0.3.2^117^ so that its extent and resolution matched that of the habitat raster. We calculated the number of people living within the AOH of each species separately, and divided this by the size of each AOH to obtain average human population density values. As living in areas with high diversity of SARSr-CoV bat hosts and large human populations may increase the likelihood of human-bat contact, viral spillover, and subsequent pathogen spread, we visualized bat species richness as well as bat species richness multiplied by human population size (which we term bat-human overlap) across the consensus map. To examine the relative importance of different habitat types to SARSr-CoV bat hosts, we calculated the proportion (by area) of each habitat type within the AOH of each species. To examine which habitat types might be most important for human exposure to bats, we calculated the number of people living in each habitat type of each species’ AOH.

### Estimating human infection with SARSr-CoVs

We used a probabilistic risk assessment to estimate SARSr-CoV spillover risk in Southeast Asia. For this analysis, we define spillover risk as the annual number of people infected by SARSr-CoVs via bat-to human transmission events. We draw on an established framework that defines risk as equal to hazard times exposure times vulnerability, where a hazard is a potential source of harm from a microbe, exposure is the likelihood of contact between humans and hazards, and vulnerability is the chance of a hazard causing harm, given exposure^37^. Here, the hazard is a bat belonging to a species known to host SARSr-CoVs, exposure is the likelihood of contact between a person and a bat, and vulnerability is the chance that a human-bat contact event results in a human infection that produces detectable antibodies.

We assumed that spillover risk can be approximated as the number of people who live in the consensus area of SARSr-CoV bat hosts (N_people_), multiplied by the probability that a human comes into contact with a bat (P_contact_), multiplied by the probability that a human-bat contact leads to a serologically detectable human infection (P_detect_), multiplied by the probability that serological detection is due to an infection within the previous year (P_pastyear_) (1).

1 $${{{{{\rm{Spillover}}}}}}\,{{{{{\rm{risk}}}}}}={{{{{{\rm{N}}}}}}}_{{{{{{\rm{people}}}}}}}* {{{{{{\rm{P}}}}}}}_{{{{{{\rm{contact}}}}}}}*{{{{{{\rm{P}}}}}}}_{{{{{{\rm{detect}}}}}}}*{{{{{{\rm{P}}}}}}}_{{{{{{\rm{pastyear}}}}}}}$$

We accounted for uncertainty (e.g., potential inaccuracy of serological tests) and variation (e.g., differences in human behavior due to gender, occupation, cultural norms) associated with the input variables by assigning a probability distribution (rather than a single fixed value) to each. We performed Latin hypercube sampling with the R package lhs v1.1.1^118^ to generate 400,000 sets of input combinations and calculated the corresponding spillover risk for each set of inputs, creating an output distribution for spillover risk. We then calculated summary statistics (median, 95% confidence interval for the median, overall range, interquartile range) for this distribution. The 95% confidence interval for the median was calculated using the MedianCI function in the R package DescTools v0.99.41^119^ with 1000 bootstrap replicates. Our choices of probability distributions for N_people_, P_contact_, P_detect_, and P_pastyear_ are described below.

For N_people_, we assigned the normal distribution $N\left(499101844,{16636728}^{2}\right)$. The mean value was derived from our calculation of the number of people living in the consensus area of SARSr-CoV bat hosts within Southeast Asia, based on 2020 WorldPop population count data. The standard deviation was chosen so that the tails of the distribution (3 standard deviations) would extend 10% beyond the mean, to reflect potential uncertainty associated with the WorldPop dataset.

To inform our choice of distribution for P_contact_, we gathered data on bat-human contacts in Southeast Asia using a systematic search of Google Scholar, PubMed, and Web of Science with the following keywords: (“bat contact” OR “human bat contact” OR “human bat interaction” OR “bat human contact” OR “bat human interaction”) AND (“Bangladesh” OR “Bhutan” OR “Brunei” OR “Cambodia” OR “China” OR “India” OR “Indonesia” OR “Lao PDR” OR “Malaysia” OR “Myanmar” OR “Nepal” OR “Philippines” OR “Singapore” OR “Sri Lanka” OR “Thailand” OR “Timor-Leste” OR “Vietnam”). English language papers published from January 1, 2000 to May 07, 2021 were included in the search. A total of 421 records were initially identified, of which 24 were retained following title and abstract screening that excluded duplicate articles, studies that only focused on bat-borne pathogens or bat ecology, and studies based outside of Southeast Asia. Full text review was performed on these 24 articles. Six articles provided data on the frequency of human contact with bats or their excreta within the last 12 months. These studies included specific data on behaviors and practices that might allow viral transmission (e.g., eating bats, receiving a bat scratch or bite, exposure to urine or guano, being in a bat cave, having bats in one’s house; summarized in Supplementary Table 3). Given that the probability of contact is bounded between 0 and 1, we assigned the beta distribution $\beta \left({{{{\mathrm{0.9366017,5.2604551}}}}}\right)$ to the 24 estimates of bat-human contact reported in the final set of six articles, using the R package fitdistrplus v1.1–3^120^ to determine the shape parameters (Supplementary Fig. 4a).

In a similar manner, to inform our choice of distribution for P_detect_, we gathered data on viral seroprevalence among people with self-reported bat contact using a systematic search of Google Scholar, PubMed, and Web of Science with the following keywords: (“bat contact” OR “human bat contact” OR “human bat interaction” OR “bat human contact” OR “bat human interaction”) AND (“serological prevalence” OR “seroprevalence” OR “serological evidence” OR “human infection” OR “spillover”). English language papers published from January 1, 2000 to June 21, 2021 were included in the search. A total of 339 records were initially identified, of which 12 were retained following title and abstract screening that excluded (i) duplicate articles, (ii) studies that reported seroprevalence in animal populations, (iii) studies that provided either seroprevalence or human-bat contact but not both, (iv) studies that detected pathogens that are unlikely to be transmitted from contact with bats, and (v) studies that analyzed human-bat contact only among identified seropositive human cases. Full text review was performed on these 12 articles. Seven articles provided data on human viral seroprevalence among populations who reported contact with bats. An additional relevant article was discovered during the revision process, making a total of eight final articles included and 16 reported estimates of seroprevalence (Supplementary Table 4). We assigned the beta distribution $\beta \left({{{{\mathrm{0.2405859,6.57698}}}}}\right)$ to these data, again using the fitdistrplus package^120^ to determine the shape parameters (Supplementary Fig. 4c). We note that it is unlikely that all known and as-yet-undiscovered bat SARSr-CoVs are likely able to infect people directly, or at all. However, the use of serology data from human surveys provides a way to account for this by providing information on exposure that has led to prior infection, albeit that the severity of that infection remains unknown.

Studies of SARS patients have provided a range of estimates for the persistence of antibodies at diagnostically detectable levels, with a maximum of six years, suggesting that the proportion of those testing seropositive could include people infected more than one year prior to testing. We note that studies of memory T cells have shown that it is possible to identify antibodies as long as 17 years after SARS infection^121^. However, our analysis uses published data on serological tests, not memory T-cell activation studies, therefore we set the maximum at six years, which is the longest time after exposure that published data for serological testing provided evidence of detectable IgG^122^. Given that our aim was to estimate the amount of bat-to-human spillover each year, we performed a calculation to estimate the likelihood that someone who tested seropositive was infected within the past year. We gathered data from papers that reported the duration of SARS-CoV immunoglobulin G (IgG) antibody detection among patients who recovered from SARS (Supplementary Table 5). Generally, the detectability of IgG rose rapidly following infection, peaked at 3–4 months following symptom onset and remained high for the first 16 months following symptom onset. Detectability then decreased over time, with ~40–70% of patients having detectable IgG after three years, and only ~10% having detectable IgG after six years. We fit a 2^nd^ order polynomial to these data, then divided the integral from 0 to 12 months by the integral from 0 to 71.5 months (when antibody detection declines to ~0 on the curve) to calculate the probability that antibody detection is due to an infection in the past year. We then repeated this process excluding the only data point at 72 months, this time dividing the integral from 0 to 12 months by the integral from 0 to 49.5 months (when antibody detection goes to ~0 on the curve). We used the two values generated from these processes to assign the uniform distribution $\beta \left({{{{\mathrm{0.232444,0.3059904}}}}}\right)$ for P_pastyear_.

Finally, we performed a global sensitivity analysis by calculating first-order and total-order Sobol sensitivity indices^123^ to understand the relative contribution of each input variable to the outcome. Sobol sensitivity indices measure the amount of variance in an outcome (here, spillover risk) due to input variables, and can be used for non-linear relationships. The first-order index represents the amount of variance in the outcome due to each input on its own. The total-order index represents the amount of variance in the outcome due to each input including interactions with other inputs. First-order and total-order indices are always ≤ 1, and first-order indices are ≤ total-order indices. All sensitivity indices were calculated using the R package sensitivity v1.25.0^124^ using the Janon-Monod method, and 95% confidence intervals were calculated using 100 bootstrap replicates using the same package^125,126^.

Sensitivity analyses indicated that P_contact_ and P_detect_ primarily contributed to variance in the outcome (see Results). Therefore, we repeated our calculation of spillover risk by excluding the three highest estimates of P_contact_ and the two highest estimates of P_detect_ found in our literature searches and refitting the distributions for these parameters. We explored three scenarios: (1) only the distribution for P_contact_ was refit, (2) only the distribution for P_detect_ was refit, and (3) both distributions were refit. See Supplementary Fig. 4 for an illustration of the original and adjusted distributions for P_contact_ and P_detect_.

### Reporting summary

Further information on research design is available in the Nature Research Reporting Summary linked to this article.

## Supplementary information

Supplementary Information

Reporting Summary

## Data Availability

Bat distribution shapefiles are available at https://www.iucnredlist.org/resources/spatial-data-download. Elevation and habitat suitability for individual species are available at https://www.iucnredlist.org/. Bat occurrence data are available at 10.15468/dl.8w26d8. Human population count data are available at 10.5258/SOTON/WP00647. Carbonate rock outcrop data are available at https://crc806db.uni-koeln.de/layer/show/296/. A global map of terrestrial habitat types (version 001) is available at 10.5281/zenodo.3666246. A global land shapefile is available at https://www.naturalearthdata.com/downloads/50m-physical-vectors/. Data from published sources are available in Supplementary Tables and also at 10.5281/zenodo.5251725.

## Code availability

All R scripts needed to reproduce the analysis and figures are available in a Github repository (https://github.com/ecohealthalliance/sars_cov_risk), also deposited in Zenodo at 10.5281/zenodo.5251725.
