# Supplementary material for: A strategy to assess spillover risk of bat SARS-related coronaviruses in Southeast Asia
Source: Nat Commun. 2022 Aug 9;13:4380. doi: 10.1038/s41467-022-31860-w (PMC9363439; doi:10.1038/s41467-022-31860-w)
Supplement: Supplementary file 1 — Supplementary Information [file 41467_2022_31860_MOESM1_ESM.pdf]

Supplementary Information for: A strategy to assess spillover risk of bat SARS-related coronaviruses in Southeast Asia

Cecilia A. Sánchez, Hongying Li, Kendra L. Phelps, Carlos Zambrana-Torrelio, Lin-Fa Wang, Peng Zhou, Zheng-Li Shi, Kevin J. Olival, Peter Daszak

Supplementary Table 1. A compiled list of bat SARSr-CoV hosts in Southeast Asia, their elevation limits, and the suitability of selected IUCN habitat types. An **X** indicates that a habitat is considered suitable for a species. Gray cell shading indicates habitats not included in a global map of terrestrial habitat types[1,2]. Elevation and suitability data were sourced from the IUCN Red List[3].

**Species**	**Family**	**Reference**	**Elevation limits (m)**		**Suitable habitat types***																			
			**Upper**	**Lower**	**1.4**	**1.5**	**1.6**	**1.9**	**3.4**	**3.5**	**3.7**	**3.8**	**4.4**	**6.0**	**7.1**	**7.2**	**8.2**	**14.1**	**14.2**	**14.3**	**14.4**	**14.5**	**14.6**	**15.8**
Aselliscus stoliczkanus	Hipposideridae	4	NA	NA											**X**									
Chaerephon plicatus	Molossidae	5	950	0		**X**	**X**								**X**	**X**			**X**		**X**			**X**
Hipposideros armiger	Hipposideridae	6	2031	100				**X**							**X**	**X**								
H. galeritus	Hipposideridae	7	1100	0		**X**	**X**							**X**	**X**	**X**								
*H. gentilis***	Hipposideridae	6	1900	0			**X**	**X**							**X**	**X**								
H. larvatus	Hipposideridae	7	2000	0			**X**	**X**							**X**	**X**								
H. pratti	Hipposideridae	6	2000	100											**X**									
Nyctalus leisleri	Vespertilionidae	8	2400	0	**X**							**X**		**X**					**X**					
Rhinolophus acuminatus	Rhinolophidae	9	1676	60			**X**								**X**									
R. affinis	Rhinolophidae	10	2000	290		**X**	**X**	**X**							**X**						**X**			
R. creaghi	Rhinolophidae	7	1500	0			**X**								**X**									
R. ferrumequinum	Rhinolophidae	7	3000	0	**X**							**X**	**X**		**X**	**X**			**X**					
R. hipposideros	Rhinolophidae	11	2000	0	**X**				**X**	**X**	**X**	**X**	**X**		**X**	**X**						**X**		
R. luctus	Rhinolophidae	12	1600	0		**X**	**X**	**X**							**X**	**X**								
R. macrotis	Rhinolophidae	4	1692	200		**X**	**X**	**X**							**X**	**X**								
R. malayanus	Rhinolophidae	13	1400	0			**X**								**X**			**X**		**X**	**X**			
R. marshalli	Rhinolophidae	14	1480	0			**X**								**X**					**X**	**X**		**X**	
R. pearsonii	Rhinolophidae	4	3077	123	**X**		**X**	**X**							**X**	**X**								
R. pusillus	Rhinolophidae	4	1370	200		**X**	**X**								**X**	**X**								
R. rex	Rhinolophidae	15	NA	NA			**X**								**X**									
R. shameli	Rhinolophidae	16	NA	NA		**X**	**X**								**X**									
R. siamensis	Rhinolophidae	12	NA	NA			**X**								**X**									
R. sinicus	Rhinolophidae	7	2769	500	**X**	**X**	**X**								**X**	**X**								
R. stheno	Rhinolophidae	17	1700	0		**X**												**X**		**X**			**X**	
R. thomasi	Rhinolophidae	6	1100	400											**X**					**X**	**X**			
Tadarida teniotis	Molossidae	18	3100	0					**X**			**X**	**X**	**X**	**X**	**X**	**X**					**X**		

*Habitat type names: 1.4. Forest -- Temperate; 1.5. Forest -- Subtropical/tropical dry; 1.6. Forest -- Subtropical/tropical moist lowland; 1.9. Forest -- Subtropical/tropical moist montane; 3.4. Shrubland -- Temperate; 3.5. Shrubland -- Subtropical/tropical dry; 3.7. Shrubland -- Subtropical/tropical high altitude; 3.8. Shrubland -- Mediterranean-type shrubby vegetation; 4.4. Grassland -- Temperate; 6.0. Rocky areas (e.g. inland cliffs, mountain peaks); 7.1. Caves and Subterranean Habitats (non-aquatic) -- Caves; 7.2. Caves and Subterranean Habitats (non-aquatic) -- Other subterranean habitat; 8.2. Desert -- Temperate; 14.1. Arable Land; 14.2. Pastureland; 14.3. Plantations; 14.4. Rural Gardens; 14.5. Urban Areas; 14.6. Subtropical/Tropical Heavily Degraded Former Forest; 15.8. Seasonally Flooded Agricultural Land

***H. gentilis* was listed as *H. pomona* in Latinne et al. 2020, but *H. gentilis* was used for our analyses due to recent taxonomic revisions; see Methods.

Supplementary Table 2. Validation of species area of habitat (AOH) using cleaned occurrence records from the Global Biodiversity Information Facility (GBIF). Species are listed in order of decreasing percent of occurrence points within a 5 km radius of the species' AOH.

**Species**	**Number of cleaned GBIF occurrence points**	**Number (%) of points within 5 km of AOH**
Rhinolophus luctus	69	63 (91)
R. creaghi	41	37 (90)
R. malayanus	52	45 (87)
R. shameli	42	35 (83)
Hipposideros galeritus	80	64 (80)
R. pusillus	76	60 (79)
H. larvatus	139	109 (78)
R. siamensis	9	7 (78)
R. affinis	189	144 (76)
R. pearsonii	95	72 (76)
Chaerephon plicatus	69	52 (75)
R. marshalli	18	12 (67)
H. gentilis	29	18 (62)
R. thomasi	41	23 (56)
Aselliscus stoliczkanus	67	36 (54)
R. ferrumequinum	2	1 (50)
H. armiger	261	124 (48)
R. acuminatus	51	20 (40)
R. macrotis	35	13 (37)
H. pratti	6	2 (33)
R. stheno	54	15 (28)
R. rex	24	4 (17)
R. sinicus	14	2 (14)
Tadarida teniotis	5	0 (0)
Nyctalus leisleri	1	0 (0)
R. hipposideros	0	NA

Supplementary Table 3. Prevalence of human-bat contact within countries in Southeast Asia. Studies were identified via a systematic literature search (see Methods in main text).

Country	Study population	Description of bat contact	Prevalence of bat contact (number reporting contact/total population)	Timeframe of contact with bat	Reference
China	People living in areas near bat populations	Bats in house	12.7% (201/1585)	Previous 12 months	[19]
		Cooked or handled bats	0.6% (9/1585)		
Indonesia	People (mostly men) living in/near a forest conservation area	Expelling	10.7% (16/150)	Not reported	[20]
		Hold/capture/hunt	46.0% (69/150)		
		Cutting	13.3% (20/150)		
		Cooking	13.3% (20/150)		
		Eating	16.7% (25/150)		
		Selling	3.3% (5/150)		
Thailand	People living in areas with high bat density	Found live bat(s) in house, community, or tourist location	20.4% (128/626)	Previous 6 months	[21]
		Consumed bat meat	15.3% (96/626)		
		Cleaned bat guano from house or the community	12.5% (78/626)		
		Found dead bat(s) in house	10.4% (65/626)		
		Bat guano mining/collecting	7.3% (46/626)		
		Cleaned bat carcasses from house or the community	7.2% (45/626)		
		Other activities (e.g. hunted bats, exposed to bat urine)	6.5% (41/626)		
		Used bat guano	4.0% (25/626)		
		Bitten by a bat	2.6% (16/626)		
	Adult guano miners, bat	Inside bat cave or roost area	57.5% (61/106)	Exposed > 5	[22]

	hunters, game wardens, residents/personnel at temples with large bat roosts	Direct bat contact (unspecified)	27.4% (29/106)	times/year	
		Bat consumption	10.4% (11/106)		
		Bat scratch	5.7% (6/106)		
		Bat bite	1.9% (2/106)		
	People living in areas where bat roosts are present	Various (hunting bats, eating bats, collecting bat guano, cleaning bat feces, finding bat carcasses in houses and communities)	46.6% (142/305)	Previous 6 months	23
Vietnam	People living in farming communities involved in raising, slaughtering, or processing wildlife or livestock	Maintained bat roosts to produce guano for fertilizer	1.2% (3/245)	Previous 12 months	24

Supplementary Table 4. Human viral seroprevalence among individuals reporting contact with bats. Studies were identified via a systematic literature search (see Methods in main text).

Country	Pathogen	Description of bat contact	Seroprevalence (number positive/total tested)	Specificity	Reference
Australia	Hendra virus (then named equine morbillivirus)	"Prolonged, significant contact" in the context of being a flying fox carer	0% (0/128)	Not reported	[25]
Cambodia	Nipah virus	Hunting bats	0% (0/4)	Not evaluated	[26]
		Palm-juice collection and selling	0% (0/15)		
Cameroon	Nipah virus	Hunting bats	3.0% (3/99)	An assay that was previously validated as having a specificity of 94–100% was further refined	[27]
		Butchering bats	4.1% (7/171)		
		General contact with bats (including hunting, butchering, and receiving bite or scratch)	3.1% (7/227)		
China	SARSr-CoV	General contact with bats	0.5% (1/199)	Not reported	[19]
	HKU10-CoV	General contact with bats	0.5% (1/199)		
	HKU9-CoV	General contact with bats	0% (0/199)		
	MERS-CoV	General contact with bats	0% (0/199)		
Lao PDR	SARS-CoV-2	Bat guano collectors	25% (3/12)	Not reported	[28] and pers. comm. with author A. Black
Malaysia	Tioman virus	Consumed fruit partially eaten by bats	6.3% (2/32)	Reported to be "highly specific"	[29]
	Nipah virus	Physical contact (unspecified)	0% (0/15)	Not reported	[30]
		Consumed fruit partially eaten by bats	0% (0/29)		
Republic of Congo	Ebola virus	General exposure to bats	14% (7/50)	Not reported, but authors used a "double immunofluorescence" protocol due to its higher specificity than regular immunofluorescence assays	[31]
		Consumption of bats	0% (0/23)		

Supplementary Table 5. Duration of SARS-CoV immunoglobulin G (IgG) antibody detection among patients who recovered from SARS.

Time post-disease onset (as originally reported)	Time post-disease onset in months (used for curve fitting)	% (number positive/total tested) with detectable IgG	Reference
0-7 days	0.25	11.8 (2/17)	[32]
8-14 days	0.5	38.5 (10/26)	[32]
15-20 days	0.75	77.3 (17/22)	[32]
21-30 days	1	91.7 (33/36)	[32]
1 month	1	100 (36/36)	[33]
1 month	1	100 (17/17)	[34]
1 month	1	100 (37/37)	[35]
31-60 days	2	93.1 (67/72)	[32]
61-90 days	3	94.3 (33/35)	[32]
3 months	3	100 (19/19)	[36]
4 months	4	100 (36/36)	[33]
4 months	4	100 (41/41)	[35]
91-120 days	4	100 (11/11)	[32]
5 months	5	100 (13/13)	[37]
7 months	7	100 (41/41)	[33]
7 months	7	100 (44/44)	[35]
121-210 days	7	100 (23/23)	[32]
10 months	10	100 (37/37)	[33]
10 months	10	100 (37/37)	[35]
12 months	12	94.7 (18/19)	[36]
1 year	12	100 (17/17)	[34]
211-365 days	12	93.9 (46/49)	[32]
16 months	16	100 (32/32)	[33]
16 months	16	100 (32/32)	[35]
18 months	18	84.2 (16/19)	[36]
20 months	20	82.4 (14/17)	[37]
24 months	24	88.2 (30/34)	[33]
24 months	24	84.2 (16/19)	[36]

24 months	24	88.6 (31/35)	[35]
366-763 days	24	89.6 (86/96)	[32]
30 months	30	80.6 (29/36)	[35]
35 months	35	84.6 (11/13)	[37]
36 months	36	74.2 (23/31)	[35]
36 months	36	42.1 (8/19)	[36]
764-1265 days	36	53.6 (15/28)	[32]
72 months	72	8.9 (2/23)	[38]

Supplementary Figure 1. Bat coronavirus research effort, as measured by publication count, in Southeast Asia. In the figure legend, tick marks from left to right indicate 0, 5, 20, 100, 150, 550, and 600 publications.

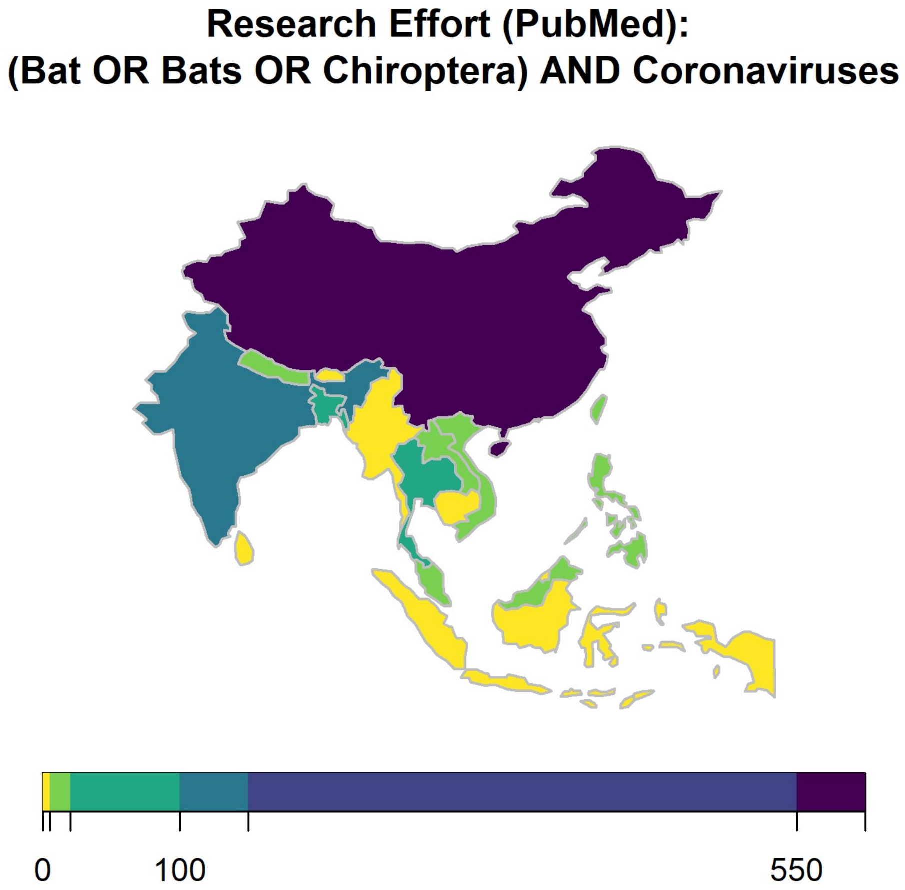

Supplementary Figure 2. Comparison of original IUCN species range sizes to AOH sizes, for Southeast Asia. Species are ordered by decreasing IUCN area.

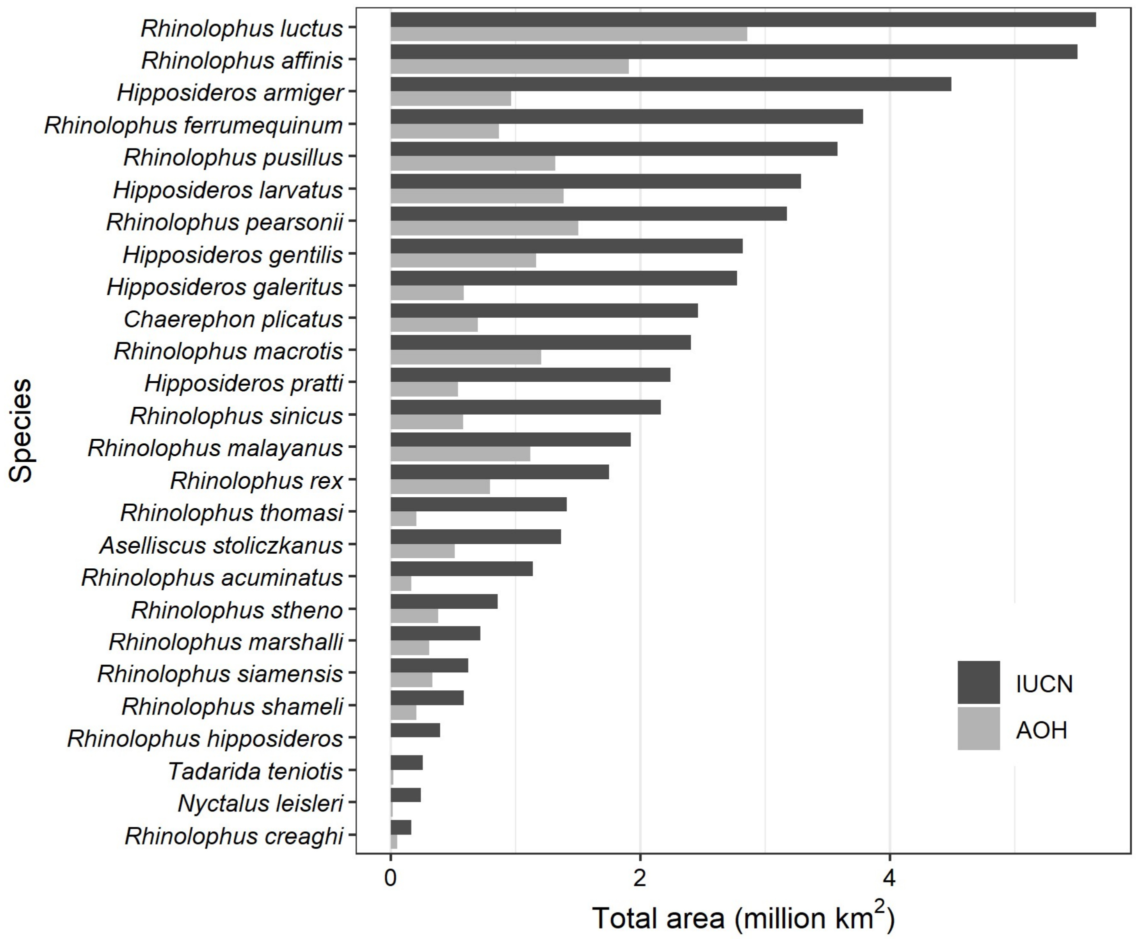

Supplementary Figure 3. Human population density per km^2 within the AOH of each SARSr-CoV bat host species.

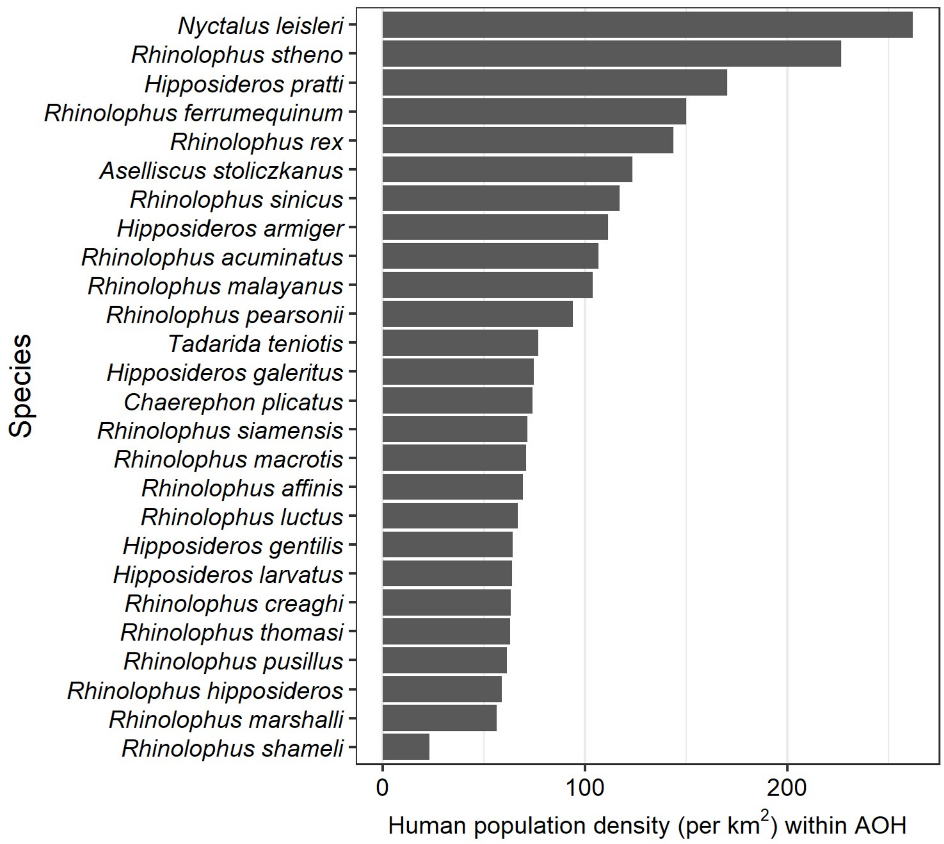

Supplementary Figure 4. Fitted beta distributions for $P_{contact}$ (the probability that a human comes into contact with a bat) and P_{detect} (the probability that a human-bat contact leads to a serologically detectable human infection). Panels **a** and **c** show the original distributions for the parameters. Panel **b** shows the re-fit $P_{contact}$ distribution after excluding the three highest estimates of human-bat contact gathered from a literature search. Panel **d** shows the re-fit P_{detect} distribution after excluding the two highest estimates of seroprevalence gathered from a literature search. The R package fitdistrplus v1.1-3[39] was used to determine the shape parameters of the distributions.

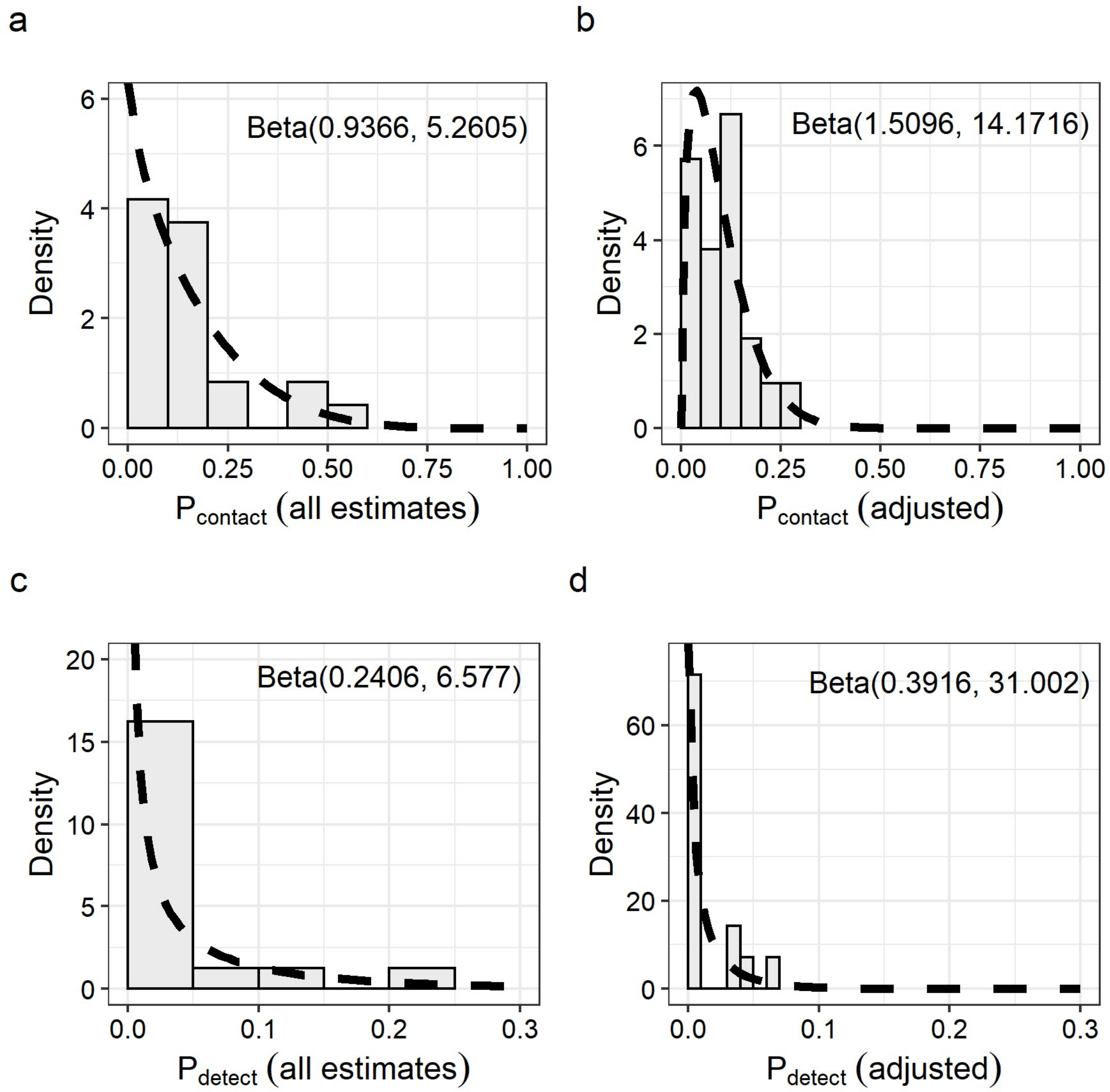

Supplementary References

1 Jung, M. *et al.* A global map of terrestrial habitat types. *Scientific Data* **7**, 256, doi:10.1038/s41597-020-00599-8 (2020).

2 Jung, M. *et al.* *A global map of terrestrial habitat types (Version 001)*, <https://doi.org/10.5281/zenodo.3666246> (2020).

3 IUCN. *The IUCN Red List of Threatened Species. Version 2021-1*, <https://www.iucnredlist.org> (2021).

4 Tao, Y. & Tong, S. Complete Genome Sequence of a Severe Acute Respiratory Syndrome-Related Coronavirus from Kenyan Bats. *Microbiology Resource Announcements* **8**, e00548-00519, doi:10.1128/MRA.00548-19 (2019).

5 Yang, L. *et al.* Novel SARS-like Betacoronaviruses in Bats, China, 2011. *Emerging Infectious Disease journal* **19**, 989, doi:10.3201/eid1906.121648 (2013).

6 Latinne, A. *et al.* Origin and cross-species transmission of bat coronaviruses in China. *Nat. Commun.* **11**, 4235, doi:10.1038/s41467-020-17687-3 (2020).

7 Anthony, S. J. *et al.* Global patterns in coronavirus diversity. *Virus Evolution* **3**, vex012, doi:10.1093/ve/vex012 (2017).

8 Drexler, J. F. *et al.* Genomic Characterization of Severe Acute Respiratory Syndrome-Related Coronavirus in European Bats and Classification of Coronaviruses Based on Partial RNA-Dependent RNA Polymerase Gene Sequences. *Journal of Virology* **84**, 11336-11349, doi:10.1128/JVI.00650-10 (2010).

9 Wacharapluesadee, S. *et al.* Evidence for SARS-CoV-2 related coronaviruses circulating in bats and pangolins in Southeast Asia. *Nat. Commun.* **12**, 972, doi:10.1038/s41467-021-21240-1 (2021).

10 He, B. *et al.* Identification of Diverse Alphacoronaviruses and Genomic Characterization of a Novel Severe Acute Respiratory Syndrome-Like Coronavirus from Bats in China. *Journal of Virology* **88**, 7070-7082, doi:10.1128/JVI.00631-14 (2014).

11 Rihtarič, D., Hostnik, P., Steyer, A., Grom, J. & Toplak, I. Identification of SARS-like coronaviruses in horseshoe bats (Rhinolophus hipposideros) in Slovenia. *Archives of Virology* **155**, 507-514, doi:10.1007/s00705-010-0612-5 (2010).

12 Wu, Z. *et al.* A comprehensive survey of bat sarbecoviruses across China for the origin tracing of SARS-CoV and SARS-CoV-2. *Research Square*, doi:10.21203/rs.3.rs-885194/v1 (2021).

13 Zhou, H. *et al.* A Novel Bat Coronavirus Closely Related to SARS-CoV-2 Contains Natural Insertions at the S1/S2 Cleavage Site of the Spike Protein. *Current Biology* **30**, 2196-2203.e2193, doi:10.1016/j.cub.2020.05.023 (2020).

14 Temmam, S. *et al.* Bat coronaviruses related to SARS-CoV-2 and infectious for human cells. *Nature* **604**, 330-336, doi:10.1038/s41586-022-04532-4 (2022).

15 Wong, A. C. P., Li, X., Lau, S. K. P. & Woo, P. C. Y. Global Epidemiology of Bat Coronaviruses. *Viruses* **11**, doi:10.3390/v11020174 (2019).

16 Delaune, D. *et al.* A novel SARS-CoV-2 related coronavirus in bats from Cambodia. *Nat. Commun.* **12**, 6563, doi:10.1038/s41467-021-26809-4 (2021).

17 Zhou, H. *et al.* Identification of novel bat coronaviruses sheds light on the evolutionary origins of SARS-CoV-2 and related viruses. *Cell* **184**, 4380-4391, doi:10.1016/j.cell.2021.06.008 (2021).

18 Lecis, R., Mucedda, M., Pidinchedda, E., Pittau, M. & Alberti, A. Molecular identification of Betacoronavirus in bats from Sardinia (Italy): first detection and phylogeny. *Virus Genes* **55**, 60-67, doi:10.1007/s11262-018-1614-8 (2019).
19 Li, H. *et al.* Human-animal interactions and bat coronavirus spillover potential among rural residents in Southern China. *Biosafety and Health* **1**, 84-90, doi:10.1016/j.bsheal.2019.10.004 (2019).
20 Basri, C. *et al.* Potential risk of viral transmission from flying foxes to domestic animals and humans on the southern coast of West Java, Indonesia. *Journal of Veterinary Medical Science* **79**, 1615-1626, doi:10.1292/jvms.17-0222 (2017).
21 Suwannarong, K. *et al.* Risk factors for bat contact and consumption behaviors in Thailand; a quantitative study. *BMC Public Health* **20**, 841, doi:10.1186/s12889-020-08968-z (2020).
22 Robertson, K. *et al.* Rabies-Related Knowledge and Practices Among Persons At Risk of Bat Exposures in Thailand. *Plos Neglect. Trop. Dis.* **5**, e1054, doi:10.1371/journal.pntd.0001054 (2011).
23 Suwannarong, K. *et al.* Bats and belief: A sequential qualitative study in Thailand. *Heliyon* **6**, doi:10.1016/j.heliyon.2020.e04208 (2020).
24 Van Cuong, N. *et al.* Rodents and Risk in the Mekong Delta of Vietnam: Seroprevalence of Selected Zoonotic Viruses in Rodents and Humans. *Vector-Borne Zoonotic Dis.* **15**, 65-72, doi:10.1089/vbz.2014.1603 (2015).
25 Selvey, L., Taylor, R., Arklay, A. & Gerrard, J. Screening of bat carers for antibodies to equine morbillivirus. *Communicable Diseases Intelligence* **20**, 477 (1996).
26 Cappelle, J. *et al.* Nipah virus circulation at human-bat interfaces, Cambodia. *Bull World Health Organ* **98**, 539-547, doi:10.2471/BLT.20.254227 (2020).
27 Pernet, O. *et al.* Evidence for henipavirus spillover into human populations in Africa. *Nat. Commun.* **5**, 5342, doi:10.1038/ncomms6342 (2014).
28 Virachith, S. *et al.* Low seroprevalence of COVID-19 in Lao PDR, late 2020. *The Lancet Regional Health – Western Pacific* **13**, 100197, doi:10.1016/j.lanwpc.2021.100197 (2021).
29 Yaiw, K. C. *et al.* Serological Evidence of Possible Human Infection with Tioman virus, a Newly Described Paramyxovirus of Bat Origin. *The Journal of infectious diseases* **196**, 884-886, doi:10.1086/520817 (2007).
30 Chong, H. T., Tan, C. T., Goh, K. J., Lam, S. K. & Chua, K. B. The risk of human Nipah virus infection directly from bats (Ptoropus hypomelanus) is low. *Neurological Journal of Southeast Asia* **8**, 31-34 (2003).
31 Moyen, N. *et al.* Risk Factors Associated with Ebola and Marburg Viruses Seroprevalence in Blood Donors in the Republic of Congo. *Plos Neglect. Trop. Dis.* **9**, e0003833, doi:10.1371/journal.pntd.0003833 (2015).
32 Wu, L.-P. *et al.* Duration of Antibody Responses after Severe Acute Respiratory Syndrome. *Emerg. Infect. Dis* **13**, 1562, doi:10.3201/eid1310.070576 (2007).
33 Liu, W. *et al.* Two-Year Prospective Study of the Humoral Immune Response of Patients with Severe Acute Respiratory Syndrome. *The Journal of infectious diseases* **193**, 792-795, doi:10.1086/500469 (2006).
34 Chang, S.-C. *et al.* Longitudinal Analysis of Severe Acute Respiratory Syndrome (SARS) Coronavirus-Specific Antibody in SARS Patients. *Clinical and Vaccine Immunology* **12**, 1455-1457, doi:10.1128/CDLI.12.12.1455-1457.2005 (2005).

35 Cao, W.-C., Liu, W., Zhang, P.-H., Zhang, F. & Richardus, J. H. Disappearance of Antibodies to SARS-Associated Coronavirus after Recovery. *New England Journal of Medicine* **357**, 1162-1163, doi:10.1056/NEJMc070348 (2007).
36 Liu, L. *et al.* Longitudinal profiles of immunoglobulin G antibodies against severe acute respiratory syndrome coronavirus components and neutralizing activities in recovered patients. *Scandinavian Journal of Infectious Diseases* **43**, 515-521, doi:10.3109/00365548.2011.560184 (2011).
37 Xie, S. Y. *et al.* [A three-year follow-up study on sera specific antibody in severe acute respiratory syndrome cases after the onset of illness]. *Zhonghua Liu Xing Bing Xue Za Zhi* **28**, 343-345 (2007).
38 Tang, F. *et al.* Lack of Peripheral Memory B Cell Responses in Recovered Patients with Severe Acute Respiratory Syndrome: A Six-Year Follow-Up Study. *The Journal of Immunology* **186**, 7264, doi:10.4049/jimmunol.0903490 (2011).
39 Delignette-Muller, M. L. & Dutang, C. fitdistrplus: An R Package for Fitting Distributions. *Journal of Statistical Software* **64**, 1-34, doi:10.18637/jss.v064.i04 (2015).
