# Supplementary material for: A strategy to assess spillover risk of bat SARS-related coronaviruses in Southeast Asia
Source: Nat Commun. 2022 Aug 9;13:4380. doi: 10.1038/s41467-022-31860-w (PMC9363439; doi:10.1038/s41467-022-31860-w)
Supplement: Supplementary file 2 — Reporting Summary [file 41467_2022_31860_MOESM2_ESM.pdf]

nature portfolio

Corresponding author(s): Peter Daszak

Last updated by author(s): May 5, 2022

Reporting Summary

Nature Portfolio wishes to improve the reproducibility of the work that we publish. This form provides structure for consistency and transparency in reporting. For further information on Nature Portfolio policies, see our Editorial Policies and the Editorial Policy Checklist.

Statistics

For all statistical analyses, confirm that the following items are present in the figure legend, table legend, main text, or Methods section.

n/a	Confirmed	
☐	☒	The exact sample size (*n*) for each experimental group/condition, given as a discrete number and unit of measurement
☒	☐	A statement on whether measurements were taken from distinct samples or whether the same sample was measured repeatedly
☒	☐	The statistical test(s) used AND whether they are one- or two-sided<br>*Only common tests should be described solely by name; describe more complex techniques in the Methods section.*
☐	☒	A description of all covariates tested
☒	☐	A description of any assumptions or corrections, such as tests of normality and adjustment for multiple comparisons
☐	☒	A full description of the statistical parameters including central tendency (e.g. means) or other basic estimates (e.g. regression coefficient) AND variation (e.g. standard deviation) or associated estimates of uncertainty (e.g. confidence intervals)
☒	☐	For null hypothesis testing, the test statistic (e.g. F, t, r) with confidence intervals, effect sizes, degrees of freedom and P value noted<br>*Give P values as exact values whenever suitable.*
☒	☐	For Bayesian analysis, information on the choice of priors and Markov chain Monte Carlo settings
☒	☐	For hierarchical and complex designs, identification of the appropriate level for tests and full reporting of outcomes
☒	☐	Estimates of effect sizes (e.g. Cohen's *d*, Pearson's *r*), indicating how they were calculated

Our web collection on statistics for biologists contains articles on many of the points above.

Software and code

Policy information about availability of computer code

Data collection	No software were used for data collection.
Data analysis	All analyses were conducted in the R statistical environment v4.0.3. The following R packages were used: CoordinateCleaner (v2.0-18), cowplot (v1.1.1), data.table (v1.13.2), DescTools (v0.99.41), fasterize (v1.0.3), fitdistrplus (v1.1-3), gdalUtils (v2.0.3.2), ggpmisc (v0.3.9), ggrepel (v0.9.1), ggspatial (v1.1.5), gitcreds (v0.1.1), gridExtra (v2.3), here (v1.0.0), lhs (v1.1.1), magrittr (v2.0.1), patchwork (v1.1.1), piggyback (v0.1.0), raster (v3.4-5), rasterVis (v0.49), RColorBrewer (v1.1-2), rentrez (v1.2.3), rgbif (v3.6.0), rgdal (v1.5-23), rnaturalearth (v0.1.0), rnaturalearthdata (v0.1.0), rredlist (v0.7.0), rworldmap (v1.3-6), scales (v1.1.1), sensitivity (v1.25.0), sf (v0.9-7), sp (v1.4-4), taxadb (v0.1.1), taxize (v0.9.99), tidyverse (v1.3.1), usethis (v2.0.0), viridis (v0.6.1), viridisLite (v0.4.0), yarrr (v0.1.5).<br><br>All R scripts needed to reproduce the analysis and figures are available in a Github repository (github.com/ecohealthalliance/sars_cov_risk), also deposited in Zenodo at https://zenodo.org/record/5251726#.YSWivEApAUE.

For manuscripts utilizing custom algorithms or software that are central to the research but not yet described in published literature, software must be made available to editors and reviewers. We strongly encourage code deposition in a community repository (e.g. GitHub). See the Nature Portfolio guidelines for submitting code & software for further information.

Data

Policy information about availability of data

All manuscripts must include a data availability statement. This statement should provide the following information, where applicable:

- Accession codes, unique identifiers, or web links for publicly available datasets
- A description of any restrictions on data availability
- For clinical datasets or third party data, please ensure that the statement adheres to our policy

Bat distribution shapefiles are available at https://www.iucnredlist.org/resources/spatial-data-download. Elevation and habitat suitability for individual species are available at https://www.iucnredlist.org/. Bat occurrence data are available at https://doi.org/10.15468/dl.8w26d8. Human population count data are available at https://dx.doi.org/10.5258/SOTON/WP00647. Carbonate rock outcrop data are available at https://crc806db.uni-koeln.de/layer/show/296/. A global map of terrestrial habitat types (version 001) is available at https://doi.org/10.5281/zenodo.3666246. A global land shapefile is available at https://www.naturalearthdata.com/downloads/50m-physical-vectors/. Compiled data from published sources are available in Supplementary Tables and also at https://zenodo.org/record/5251726#.YSWivEApAUE.

Field-specific reporting

Please select the one below that is the best fit for your research. If you are not sure, read the appropriate sections before making your selection.

☐ Life sciences ☐ Behavioural & social sciences ☒ Ecological, evolutionary & environmental sciences

For a reference copy of the document with all sections, see nature.com/documents/nr-reporting-summary-flat.pdf

Ecological, evolutionary & environmental sciences study design

All studies must disclose on these points even when the disclosure is negative.

Study description	We used species distribution data, habitat preference data, and elevation data to estimate the richness and distribution (restricted to Southeast Asia) of bat species that host SARSr-CoVs. We assessed overlap between bat host species distribution and human distribution. We used a probabilistic risk assessment to estimate the annual number of people infected by SARSr-CoVs via bat-to-human transmission events in Southeast Asia, using information gathered from the literature on human-bat contacts, viral seroprevalence among humans reporting bat contact, and human SARS antibody duration. We performed sensitivity analyses to assess the contribution of input variables to the outcome variable.
Research sample	We performed 400,000 simulations to estimate the annual number of people infected by SARSr-CoVs via bat-to-human transmission events in Southeast Asia. The number of simulations was chosen to ensure that sensitivity indices were >1.
Sampling strategy	Not applicable (no sampling to collect data).
Data collection	No primary data were collected for this study. Previously published data on the probability that a human comes into contact with a bat, the probability that a human-bat contact leads to a serologically detectable human infection, and human SARS seroprevalence were gathered via literature searches performed by author H.L. and compiled in .csv files.
Timing and spatial scale	The spatial scale of the study was Southeast Asia, which we defined as the following countries and administrative regions: Bangladesh, Bhutan, Brunei, Cambodia, China, Hong Kong SAR, Macao SAR, India, Indonesia, Lao People's Democratic Republic, Malaysia, Myanmar, Nepal, the Philippines, Singapore, Sri Lanka, Thailand, Timor-Leste, and Vietnam. Computer simulations were performed in 2021.
Data exclusions	Based on reviewer feedback that was skeptical of high estimates of Pcontact and Pdetect gathered from the literature, we repeated our calculation of spillover risk by excluding the three highest estimates of Pcontact and the two highest estimates of Pdetect found in our literature searches and refitting the distributions for these parameters. We explored three scenarios: 1) only the distribution for Pcontact was refit, 2) only the distribution for Pdetect was refit, and 3) both distributions were refit. Exclusion criteria were chosen in response to reviewer comments, rather than pre-established.
Reproducibility	Our analysis is reproducible with the files on the Zenodo and Github repositories.
Randomization	Not applicable (no experimental trials undertaken in the study).
Blinding	Not applicable (no experimental trials undertaken in the study).

Did the study involve field work? ☐ Yes ☒ No

Reporting for specific materials, systems and methods

We require information from authors about some types of materials, experimental systems and methods used in many studies. Here, indicate whether each material, system or method listed is relevant to your study. If you are not sure if a list item applies to your research, read the appropriate section before selecting a response.

Materials & experimental systems

n/a	Involved in the study	
[x]	[ ]	Antibodies
[x]	[ ]	Eukaryotic cell lines
[x]	[ ]	Palaeontology and archaeology
[x]	[ ]	Animals and other organisms
[x]	[ ]	Human research participants
[x]	[ ]	Clinical data
[x]	[ ]	Dual use research of concern

Methods

n/a	Involved in the study	
[x]	[ ]	ChIP-seq
[x]	[ ]	Flow cytometry
[x]	[ ]	MRI-based neuroimaging
